# Development of a simple and specific direct competitive ELISA for the determination of artesunate using an anti-artesunate polyclonal antiserum

**DOI:** 10.1186/s41182-016-0037-2

**Published:** 2016-11-21

**Authors:** Yoshinori Mitsui

**Affiliations:** Department of Parasitology, Institute of Tropical Medicine, Nagasaki University, 1-12-4 Sakamoto, Nagasaki, 852-8523 Japan

**Keywords:** Artesunate, ELISA, Malaria, Polyclonal antibody

## Abstract

**Background:**

Since artesunate (ART) became a vital component of artemisinin (ARM)-based combination therapies for the treatment for malaria, counterfeit ART drugs have spread in regions of Southeast Asia and Africa. The consumption of counterfeit ART drugs has resulted in the death of many patients. Thus, evaluating the quality of ART drugs is needed. There are several methods for quantitating the ART content in tablets, the most common being a high-performance liquid chromatography. However, that method is hampered by the need for expensive equipment and a rather time-consuming process of extraction. By contrast, enzyme-linked immunosorbent assays (ELISAs) are faster and much less expensive, and they require less sample preparation than the above method. The objective of the present study was to establish a simple and specific direct competitive ELISA for the determination of ART concentrations using an anti-ART polyclonal antibody (pAb).

**Results:**

Anti-ART pAb was raised in mice, and ART-horseradish peroxidase (HRP) conjugate was produced. A direct competitive ELISA was performed by simultaneously incubating ART and the ART-HRP conjugate with the anti-ART pAb over a second antibody. Subsequently, the enzyme activity of the remaining ART-HRP conjugate was measured. The intra- and inter-assay coefficients of variation of the ELISA were less than 10 % in the range of 0.3 to 30 ng/ml with a detection limit of 0.1 ng/ml. The cross-reactivities of the anti-ART pAb with ARM and dihydroartemisinin were 0.12 and 0.04 %, respectively, and those with other antimalarial drugs were negligible. Furthermore, the recovery of 10 or 50 ng/ml ART added to the drug tablet solutions containing an expected amount of 10 ng/ml was estimated by the ELISA. The recovery of the ART amount ranged between 98 and 106 %, with coefficient variations of less than 7.0 %.

**Conclusions:**

The present ELISA is a simple and specific method for the determination of ART concentrations. Thus, this ELISA can be used to identify ART counterfeits and substandard drugs and to quantify the ART drugs.

## Background

Since WHO recommended artemisinin (ARM)-based combination therapies (ACTs) as the first-line treatment for malaria [1], artesunate (ART) has been a vital component of ACTs and widely used in the treatment of malaria. On the other hand, the massive demand for and relatively high retail price of ART have promoted the spread of counterfeit ART drugs in regions of Southeast Asia and Africa [2–4].

ART is currently the target of an extremely sophisticated and prolific counterfeit drug trade, both in terms of ART tablets and packaging [5–7], the latter closely resembling that of the authentic product. The counterfeit or substandard ART drugs can cause therapeutic failure and death in malaria patients and increase the risk of selecting and spreading of malarial parasites resistant to artemisinin derivatives [7, 8].

In response to the threat of counterfeit ART, Green et al. developed and validated a rapid and inexpensive colorimetric Fast Red TR dye test to distinguish counterfeit ART from genuine tablets [9]. However, counterfeiters sometimes include subtherapeutic amounts of ART in the tablets in order to evade the rapid colorimetric tests currently used to detect counterfeits [7, 10, 11].

Generally, high-performance liquid chromatography (HPLC) is the standard analytical method to assess a drug quantitatively and accurately differentiate between genuine and counterfeit ART drugs [12, 13]. However, the procedure involves a time-consuming extraction and is unsuitable for the measurement of large sample numbers. In addition, resources to purchase and maintain such expensive equipment are not always available in many tropical and subtropical countries. By contrast, enzyme-linked immunosorbent assays (ELISAs) are generally very simple and specific methods for quantitative measurements of drugs. Ferreira and Janick and Tanaka et al. produced polyclonal antibodies (pAbs) and monoclonal antibodies (mAbs) to ART [14, 15], respectively, and developed an indirect competitive ELISA for detection of ARM in botanical samples. Subsequently, Wang et al. developed an indirect competitive ELISA of ART using anti-ART mAb produced by He et al. and applied the assay for the quantification of ART tablets [16, 17]. The assay might lack adequate specificity to ART, however, because of the cross-reactivities of the anti-ART mAb with ARM (15.5 %) and dihydroartemisinin (DHA) (8.8 %). The objective of the present study was to produce an anti-ART pAb specific for ART and to develop a simple and specific direct competitive ELISA for detecting ART amounts using the anti-ART pAb.

The present study was conducted for (1) the production of the anti-ART pAbs specific to ART in mice, (2) the establishment of a direct competitive ELISA for ART, and (3) the assessment of the recovery of ART added to tablet sample solutions estimated by the ELISA.

## Methods

### Chemicals

ART and DHA were obtained from LKT Laboratories, Inc. (St. Paul, MN, USA) and Toronto Research Chemicals Inc. (North York, ON, Canada), respectively. Artesunat® tablets (Traphaco Joint Stock Company, Hanoi, Vietnam) were purchased from a local pharmacy in Hanoi, Vietnam. 1-Ethyl-3(3-diethylaminopropyl) carbodiimide hydrochloride (EDC) was purchased from Dojindo Laboratories (Kumamoto, Japan). Horseradish peroxidase (HRP) was obtained from Roche Diagnostics GmbH (Mannheim, Germany). Bovine serum albumin (BSA), ARM, chloroquine (CQ)·2H_3_PO_4_, primaquine (PQ)·2H_3_PO_4_, mefloquine (MQ)·HCl, pyrimethamine (PY), and sodium sulfadiazine (SF) were purchased from Sigma (St. Louis, MO, USA). Quinine (QN) and 3,3′,5,5′-tetramethylbenzidine (TMB) were obtained from Wako Pure Chemical Industries, Ltd. (Osaka, Japan). Amodiaquine (AQ)·2HCl and goat anti-mouse IgG(Fc) antibody were purchased from MP Biomedicals Inc. (Fountain Parkway Solon, OH, USA). All other chemicals and solvents were reagent grade or chemically pure.

### Preparation of ART-BSA conjugate used as an immunogen

The ART-BSA conjugate used as an immunogen was prepared according to a modified *N*-hydroxysuccinimide (NHS) ester method [18]. EDC (5 mg) and NHS (5 mg) were added to a solution of ART (5 mg) dissolved in 0.3 ml of a solution of dimethylformamide (DMF)/0.1 M sodium phosphate buffer solution (PB), pH 7.0 (2/1, *v*/*v*), and then stirred for 1.5 h at room temperature (rt). The result was slowly added to a solution of BSA (5 mg) dissolved in 0.3 ml of 0.1 M PB (pH 7.2) and vigorously stirred for 1.5 at rt. The conjugate was then dialyzed against 2 l of phosphate-buffered saline (PBS) (137 mM NaCl, 10 mM Na_2_HPO_4_, 2.7 mM KCl, 1.8 mM KH_2_PO_4_, pH 7.4) and stored at −30 °C.

### Preparation of an anti-ART polyclonal antiserum

Five female ICR mice aged 6 weeks (Clea Japan, Inc., Tokyo, Japan) were injected intraperitoneally with 20 μl of a saline solution containing 20 μg of ART-BSA conjugate emulsified with 180 μl of Freund’s complete adjuvant at weeks 0, 4, and 6. At week 8, the blood was drawn from each mouse by heart puncture and pooled, and the serum was separated by centrifugation and stored at −80 °C until use.

### Preparation of ART-HRP conjugate

The ART-HRP conjugate was also prepared by the NHS method. EDC (10 mg) and NHS (5 mg) were added to a solution of ART (10 mg) dissolved in a 0.3 ml of a solution of DMF/PB (pH 7.0) (2/1, *v*/*v*) and then stirred for 1 h at rt. The result was slowly added to a solution of HRP (5 mg) dissolved in 0.35 ml of PB (pH 7.2) and vigorously stirred for 3 h at rt. After centrifugation at 200*g* for 2 min, the supernatant solution was dialyzed against 2 l PBS overnight at rt. The final solution was stored with 20 % ethylene glycol at −80 °C.

### A direct competitive ELISA for the determination of ART concentrations using an anti-ART polyclonal antiserum

**Fig. 1:**
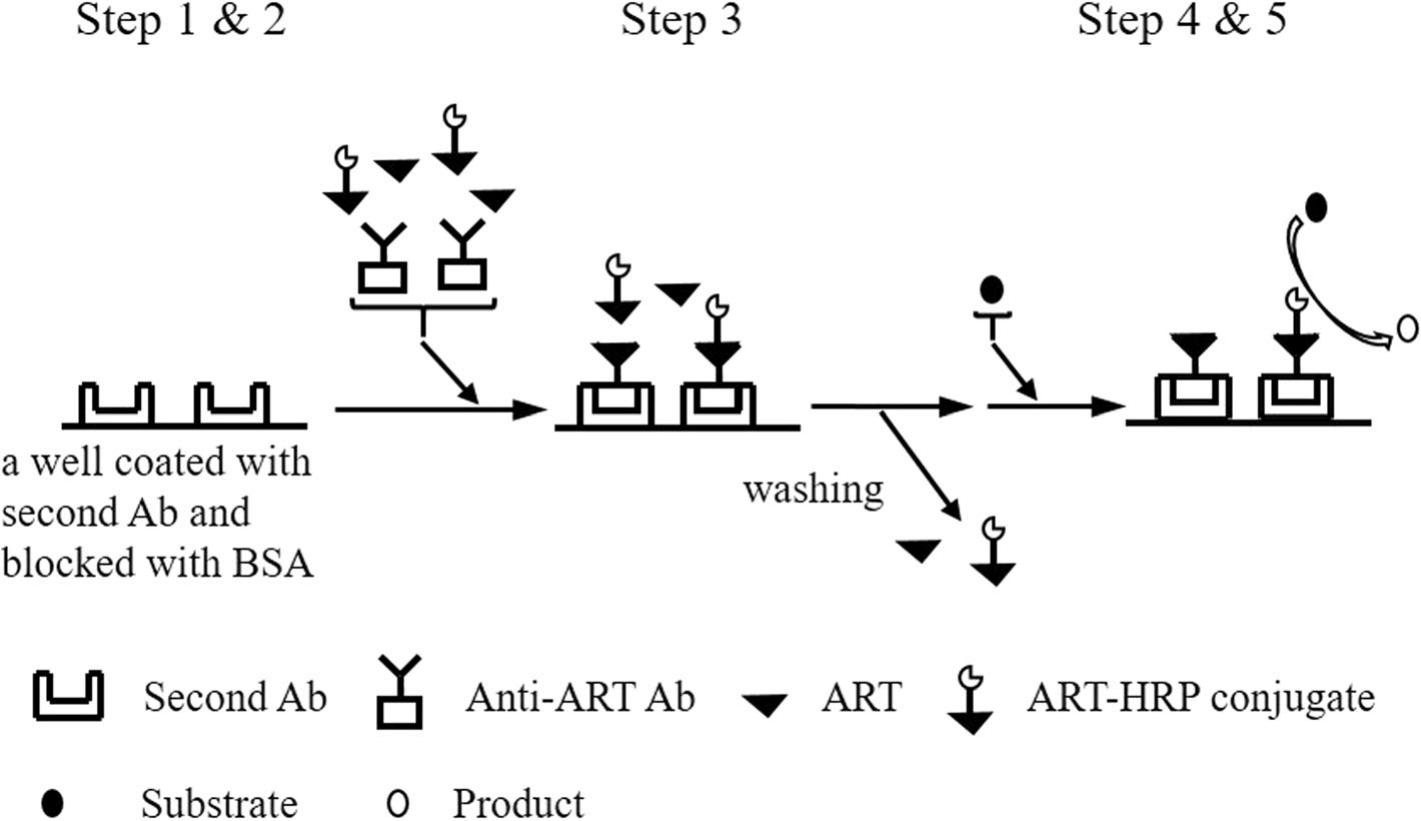
Schematic illustration for the direct competitive ELISA of artesunate

As shown in Fig. 1, the assay was carried out in the following steps. Step 1: each well of a 96-well microtiter plate (Immunoplate Maxisorp, Nunc, Roskilde, Denmark) was coated with 200 μl of goat anti-mouse IgG(Fc) antibody (3.5 μg/ml) diluted with 0.1 M sodium bicarbonate buffer (pH 9.6). The plate was then incubated for 1 h at rt, and the antibody solution was discarded. Step 2: 300 μl of PBS containing 1 % BSA was added to each well, and the plate was incubated for 1 h at rt. The blocking solution was discarded. Step 3: after washing with 300 μl of PBS containing 0.05 % Tween 20 (PBS-T), 100 μl of standard ART or tested sample diluted in PBS containing 0.1 % BSA (BSA-PBS), 50 μl of 1:20,000-diluted stored solution of ART-HRP conjugate in BSA-PBS, and 50 μl of 1:10,000-diluted anti-ART antiserum in BSA-PBS were added to each well. Subsequently, the incubation was continued overnight at 4 °C. The wells were then washed three times with 300 μl of PBS-T. Step 4: 200 μl of a substrate solution (20 ml of 0.1 M sodium acetate buffer (pH 5.0) supplemented with 200 μl of dimethyl sulfoxide containing 10 mg/ml TMB and 10 μl of 30 % hydrogen peroxide) was added to each well and incubated for 1 h at rt. Step 5: the reaction was terminated by adding 100 μl of 1 M phosphoric acid, and the absorbance was measured at 450 nm with a microplate reader (Multiskan Bichromatic; Labsystems, Helsinki, Finland).

### Specificity of anti-ART pAb to ART-related antimalarial drugs

Cross-reactivity (CR) of the anti-ART pAb to ART-related antimalarial drugs was tested by the competitive ELISA. ART, ARM, DHA, QN, CQ, PQ, MQ, AQ, PY, and SF were used as an inhibitor replacing ART. Each drug was dissolved in BSA-PBS or BSA-PBS containing 0.5 % DMF to a concentration of 10 μg/ml and diluted serially in BSA-PBS. Concentrations of these solutions were estimated using the competitive ELISA. CRs of anti-ART pAb for ART-related antimalarial drugs were determined by dividing the 50 % inhibitory concentration (IC_50_) of ART by the IC_50_ values of each antimalarial drug and expressed as a percentage.

### Intra- and inter-assays

In intra-assay, five replicate samples containing ART at six different concentrations of 0.1, 0.3, 1.0, 3.0, 10, and 30 ng/ml were prepared and amounts of the ART in each sample were estimated using the described competitive ELISA procedure. Mean and standard deviation (SD) in six different concentrations were calculated, thereafter. The coefficient of variation (CV) was calculated using formula: 100 × SD/mean. Furthermore, the recovery was calculated using formula: 100 × mean estimated amounts/added amounts. In inter-assay, other five replicate samples containing ART at six different concentrations of 0.1, 0.3, 1.0, 3.0, 10, and 30 ng/ml were also prepared 1 week later, and amounts of the ART in each sample were estimated using the competitive ELISA. After adding data of the five replicates in the intra-assay, amounts of ART estimated in 10 replicate samples were combined, and mean, SD, CV, and the recovery were calculated.

### Recovery of ART added to tablet sample solutions estimated by the ELISA

One tablet of Artesunat® (each tablet was expected to contain 50 mg ART; one tablet weighed around 260 mg) was crushed with a mortar and pestle. One twentieth of the crushed powder (expected to contain 2.5 mg of active ART) was weighed accurately and suspended in 1 ml of methanol/deionized water (DW) (1/1, *v*/*v*). To a 1.5-ml test tube, 100 μl of the suspended solution and 0, 100, or 500 μl stock solution of 2.5 mg/ml ART (dissolved in methanol) were added, and the volume was adjusted to 1 ml with 0.1 % BSA-PBS. The solution was further diluted up to 25,000-fold with 0.1 % BSA-PBS at final concentrations of 0, 10, or 50 ng/ml of added ART. The concentrations of ART were estimated by the direct competitive ELISA as described above.

In intra-assay, six replicate samples of tablet sample solution alone, supplemented with 10 or 50 ng/ml ART, were prepared and amounts of ART in each replicate samples were estimated using the described competitive ELISA. Mean and SD of amounts of ART estimated in each replicate samples were calculated, thereafter. The CV was calculated using formula: 100 × SD/mean. Furthermore, the recovery was calculated using formula: 100 × (mean ART amounts estimated − mean estimated ART amounts in a tablet sample solution alone)/ART amounts added. In inter-assay, other six replicate samples of tablet sample solution alone, supplemented with 10 or 50 ng/ml ART, were also prepared 1 week later, and amounts of ART in each replicate samples were estimated using the ELISA. After adding data of the six replicates in the intra-assay, amounts of ART estimated in 12 replicate samples were combined, and mean, SD, CV, and the recovery were calculated.

## Results

### Standard curve of the direct competitive ELISA for ART

**Fig. 2:**
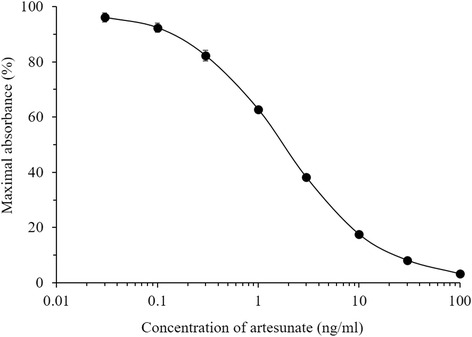
Standard curve for the direct completive ELISA of artesunate. Each point is the mean ± standard deviation of six assays

The standard curve of the direct competitive ELISA for ART is shown in Fig. 2. The dose response range yielded was between 0.03 and 100 ng/ml.

### Specificity of the anti-ART pAb

Specificities of the anti-ART pAbs with ART derivatives and current antimalarial drugs are shown in Table 1. The cross-reactivities of the anti-ART pAbs with ARM and DHA were 0.12 and 0.04 %, respectively, whereas those with all other antimalarial drugs were less than 0.023 %.

**Table 1:** Cross-reactivities of anti-artesunate (ART) polyclonal antibodies with ART and related antimalarial drugs

ART and related antimalarial drugs	50 % inhibitory concentration^b^ (ng/ml)	Cross-reactivity^c^ (%)
ART	2.3	100
Artemisinin^a^	2000	0.12
Dihydroartemisinin^a^	5200	0.04
Quinine^a^	>10^4^	<0.023
Chloroquine	>10^4^	<0.023
Primaquine	>10^4^	<0.023
Mefloquine^a^	>10^4^	<0.023
Amodiaquine	>10^4^	<0.023
Pyrimethamine^a^	>10^4^	<0.023
Sulfadiazine	>10^4^	<0.023

^a^Test compound solutions contained 0.5 % dimethylformamide at a concentration of 10^4^ ng/ml

^b^Concentrations of test compounds were expressed as free base

^c^Cross-reactivity of anti-ART antibodies for ART-related antimalarial drugs was determined by dividing the 50 % inhibitory concentration (IC_50_) of ART by the IC_50_ values of each antimalarial drug and expressed as a percentage

### Precision and accuracy of the direct competitive ELISA of ART

The precision and accuracy of the ELISA was examined at six different ART concentrations over the range of 0.1–30 ng/ml (Table 2). The recovery of added ART ranged between 97 and 106 % in the intra- and inter-assays. The coefficients of variation were less than 7 % in the range of 0.3–30 ng/ml. The detection limit of the ELISA was 0.1 ng/ml.

**Table 2:** Precision and accuracy of a direct competitive ELISA of artesunate (ART)

Sample	Added ART (ng/ml)	Estimated ART^a^ (ng/ml)	Coefficient of variation^b^ (%)	Recovery^c^ (%)
Intra-assay (*n* = 5)	0.1	0.10 ± 0.03	25.5	106.0
	0.3	0.30 ± 0.01	3.9	99.0
	1.0	1.00 ± 0.02	2.3	99.6
	3.0	2.9 ± 0.1	2.9	97.3
	10.0	10.1 ± 0.2	2.4	100.8
	30.0	29.3 ± 1.7	5.9	97.7
Inter-assay (*n* = 10)	0.1	0.10 ± 0.02	22.0	103.0
	0.3	0.30 ± 0.01	4.3	99.3
	1.0	1.00 ± 0.02	2.4	99.9
	3.0	3.0 ± 0.1	2.7	98.7
	10.0	10.1 ± 0.3	3.2	100.9
	30.0	29.2 ± 1.8	6.3	97.2

*n* number of determinations

^a^Mean ± standard deviation

^b^Coefficient of variation = 100 × standard deviation/mean

^c^Recovery (%) = 100 × (mean estimated amounts/added amounts)

### Recovery of ART added to a tablet sample solution estimated by the ELISA

The recovery of ART added to ART tablet sample solutions is shown in Table 3. The estimated concentration of active ART in the tablet sample solutions was 7.6 ng/ml in intra- and inter-assays. Since a concentration of 10 ng/ml was equivalent to 50 mg per one tablet, the estimated amount of ART in the tablet samples was 38.0 mg (=50 × 7.6/10, active ART in the tablet 76 %). When 10 or 50 ng/ml ART was added to the tablet sample solutions, the recovery of the ART ranged between 98 and 106 % with a coefficient of variation of less than 7 % in the intra- and inter-assays.

**Table 3:** Recovery of artesunate (ART) added to tablet sample solutions estimated by the ELISA

Sample	Added ART (ng/ml)	Estimated ART^a^ (ng/ml)	Coefficient of variation^b^ (%)	Recovery^c^ (%)
Intra-assay (*n* = 6)	0	7.6 ± 0.4	5.2	–
	10	17.5 ± 0.8	4.8	99.0
	50	56.8 ± 2.3	4.1	98.3
Inter-assay (*n* = 12)	0	7.6 ± 0.4	5.1	–
	10	18.2 ± 1.3	7.0	105.7
	50	57.8 ± 2.2	3.8	100.3

*n* number of determinations

^a^Mean ± standard deviation, a concentration of 10 ng/ml is equivalent to 50 mg of a tablet sample

^b^Coefficient of variation = 100 × standard deviation/mean

^c^Recovery (%) = 100 × (mean ART amounts estimated − mean estimated ART amounts in a tablet sample solution alone)/ART amounts added

## Discussion

A direct competitive ELISA of diethylcarbamazine (DEC) was previously reported to be more simple, sensitive, and reproductive than an indirect competitive ELISA of DEC [19, 20]. Thus, the present study was an attempt to develop a direct competitive ELISA of ART.

As a prerequisite for the development of the direct competitive ELISA of ART, ART-BSA and ART-HRP conjugates were required. To obtain ART-BSA conjugate as the immunogen of ART, Tanaka et al. simultaneously incubated ART, BSA, and EDC [15]. However, this method had the disadvantage that self-coupling of the BSA molecules might occur. In order to avoid the undesirable self-coupling of BSA or HRP, the NHS ester method was applied for the conjugation of ART with BSA or HRP in the present study [18]. The ART-BSA conjugate as an immunogen of ART was successfully prepared and used to elicit anti-ART pAbs specific for ART. Consequently, the anti-ART pAbs and the ART-HRP conjugate facilitated the development of a direct competitive ELISA of ART.

The cross-reactivities of the anti-ART pAbs with ARM and DHA produced in the present study were 0.12 and 0.04 %, respectively, while those with other antimalarial drugs were less than 0.023 %. These results suggest that the anti-ART pAbs strongly recognize the entire structure of the ART molecule. On the other hand, the cross-reactivities of the anti-ART mAbs produced by Tanaka et al. and He et al. were 15.9 and 15.5 % with ARM and 4.7 and 8.8 % with DHA [15, 16], respectively. The present anti-ART pAbs had a higher specificity to ART than the anti-ART mAbs of either Tanaka et al. or He et al. Thus, the present assay is considered to be more specific for the determination of ART than the assay of Tanaka et al. and Wang et al. [15, 17].

Tanaka et al. showed that the detection limit for ART in an indirect competitive ELISA using an anti-ART mAb was around 2 ng/ml [15]. On the other hand, the present ELISA showed that the detection limit for ART was 0.1 ng/ml. The present ELISA is clearly more sensitive for the determination of ART concentration than the indirect ELISA of Tanaka et al. (2007) [15]. In addition, the recovery of ART in intra- and inter assays was 97–101 % with less than 7 % of coefficients of variation in the range of 0.3 to 30 ng/ml. The results demonstrated the precise and accurate of the present ELISA.

Subsequently, the recovery of amounts of ART added to tablet sample solutions estimated by the ELISA was validated. The estimated amount of active ART in drug tablet samples was 38.0 mg (recovery rate 76 %) in the intra- and inter-assays, which was lower than the 50 mg expected in the drug tablets (Table 3). The lower estimation is probably affected by various excipients such as lactose, starch, and cellulose in the drug tablets. However, the recovery of amounts of ART added to the ART tablet sample solutions was close to 100 %. The lower estimation of active ART in the drug tablets is unlikely due to interference of the excipients. As the assay was conducted 1 month before the expiry date of the drug tablets, the lower estimation of active ART in the drug tablets may be due to degradation and/or loss of active ART under the conditions of high humidity/high room temperature during long storage and transport.

Counterfeit or substandard ART drugs have led to death in malaria patients [7] and allow the selection and spread of malarial parasites resistant to artemisinin derivatives [8, 21]. Thus, it is important to quantify the amount of active ART in drug tablets. HPLC methods are currently used to estimate the amount of active ART [22]. However, the use of methods requiring expensive equipment and trained technicians is restricted to well-equipped laboratories and thereafter is unfeasible in many malaria-endemic areas where quantification of the amount of ART in tablet samples is needed. By contrast, the present ELISA method is simple, specific, accurate, precise, and less time-consuming than the HPLC methods.

## Conclusions

In the present study, the simple and specific ELISA for quantitative determination of ART was established. The ELISA method will definitely contribute to not only to the identification of counterfeit ART drugs but also to the quantification of the active ART present in the drug tablets and thus prevent the proliferation of poor quality ART tablets. Furthermore, the intra- and inter-assay CVs of the ELISA were less than 10 % in the range of 0.3 to 30 ng/ml with a detection limit of 0.1 ng/ml. The ELISA allows to accurately measure a low concentration of ART in biological fluids. Therefore, the ELISA is applicable for the determination of ART concentrations in blood and can contribute to pharmacokinetic studies of the ART in humans and animals.

## Abbreviations

ACT: Artemisinin-based combination therapy
AQ: Amodiaquine
ARM: Artemisinin
ART: Artesunate
BSA: Bovine serum albumin
BSA-PBS: PBS containing 0.1 % BSA
CQ: Chloroquine
CR: Cross-reactivity
CV: Coefficient of variation
DEC: Diethylcarbamazine
DHA: Dihydroartemisinin
DMF: Dimethylformamide
DW: Deionized water
EDC: 1-Ethyl-3(3-diethylaminopropyl) carbodiimide hydrochloride
ELISA: Enzyme-linked immunosorbent assay
HPLC: High-performance liquid chromatography
HRP: Horseradish peroxidase
IC_50_: 50 % inhibitory concentration
mAb: Monoclonal antibody
MQ: Mefloquine
NHS: *N*-Hydroxysuccinimide
pAb: Polyclonal antibody
PB: Sodium phosphate buffer solution
PBS: Phosphate-buffered saline
PBS-T: PBS containing 0.05 % Tween 20
PQ: Primaquine
PY: Pyrimethamine
QN: Quinine
rt: Room temperature
SD: Standard deviation
SF: Sulfadiazine
TMB: 3,3′,5,5′-Tetramethylbenzidine
